# X-linked hypophosphatemic rickets: case report with perspectives and challenges

**DOI:** 10.31744/einstein_journal/2026RC1788

**Published:** 2026-09-09

**Authors:** Beatriz Almeida Ribeiro, Henrique Massashi Soga, Fernanda Teresa de Lima, Amancio Ramalho, Cintia Giunchetti Strabelli Nascimento, Eduardo Juan Troster, Teresa Cristina Gomes Alfinito Vieira

**Affiliations:** 1 Hospital Israelita Albert Einstein Faculdade Israelita de Ciências da Saúde Albert Einstein São Paulo SP Brazil Faculdade Israelita de Ciências da Saúde Albert Einstein, Hospital Israelita Albert Einstein, São Paulo, SP, Brazil.; 2 Hospital Israelita Albert Einstein São Paulo SP Brazil Hospital Israelita Albert Einstein, São Paulo, SP, Brazil.

**Keywords:** Rickets, Familial hypophosphatemic rickets, Antibodies, monoclonal, Genetic diseases, X-linked, Bone diseases, metabolic

## Abstract

X-linked dominant hypophosphatemic rickets (XLH) is a rare genetic disorder caused by mutations in the *PHEX* gene and characterized by chronic hypophosphatemia due to dysregulation of fibroblast growth factor 23 (FGF23). This condition leads to bone deformities, growth impairment, and chronic pain. Conventional treatment with oral phosphate and calcitriol often has limited efficacy and is associated with adverse effects such as hyperparathyroidism and nephrocalcinosis. This case report describes the therapeutic outcomes of three pediatric patients with XLH treated with burosumab, a monoclonal antibody targeting FGF23. Burosumab acts directly on the underlying pathophysiology of the disease, improving phosphate homeostasis and bone metabolism. The patients showed improvements in clinical symptoms and biochemical parameters, with fewer adverse effects and greater treatment adherence compared with traditional therapy. These findings highlight burosumab as a promising and potentially more effective alternative for managing XLH in pediatric patients.

## INTRODUCTION

Rickets is a systemic disease that occurs during childhood growth and results from abnormal differentiation and maturation of chondrocytes with abnormal mineralization of the growth plate and bone deformities. Its etiology is heterogeneous, including vitamin D deficiency and rare hereditary forms. Although the clinical presentation may be similar, the molecular etiology and biochemical mechanisms that lead to bone deformities are distinct.^(1)^

X-linked dominant hypophosphatemic rickets (XLH) is one of the rare hereditary forms of genetic rickets with a prevalence of 1:20,000 individuals.^(2)^ It is caused by inactivating mutations in the X-linked phosphate-regulating endopeptidase homolog (*PHEX*) gene, located in chromosomal region Xp21.11.

The *PHEX* gene encodes the PHEX protein, an endopeptidase involved in regulating serum levels of fibroblast growth factor 23 (FGF23), a bone-derived hormone that suppresses renal phosphate reabsorption and inhibits the synthesis of 1,25-dihydroxyvitamin D (1,25(OH)^2^D), commonly referred to as calcitriol. Although the exact physiologic mechanisms are not yet fully understood, PHEX activity appears to reduce FGF23 expression, thereby increasing the expression of renal sodium-phosphate cotransporters (NaPi-IIa and NaPi-IIc) in the proximal tubules and enhancing inorganic phosphate (Pi) reabsorption.^(3)^ It is important to note that most conventional assays used to measure vitamin D metabolites do not differentiate between vitamin D_2_ and D_3_ derivatives; liquid chromatography–tandem mass spectrometry (LC-MS/MS) is a notable exception. Consequently, circulating 1,25(OH)_2_D concentrations are typically reported without specification of the D_3_ isoform.

In XLH, loss of function of the PHEX protein leads to overexpression of FGF23, which drastically reduces renal Pi reabsorption, leading to significant phosphate loss through the kidneys, chronic hypophosphatemia, and low calcitriol levels.^(4)^ Since Pi is a component of hydroxyapatite, its prolonged deprivation affects bone mineralization. In pediatric patients with XLH, bone mineral deficiency and rickets cause bowed legs and disproportionate short stature due to growth plate alterations, as well as deformities in long bones and the spine. Bone pain is a common symptom, affecting 65% and 80% of pediatric patients with XLH, significantly impairing their quality of life.^(5)^ Additionally, low dental mineralization associated with XLH can lead to frequent endodontic infections. Dental symptoms do not appear to be caused by FGF23 but rather by the direct local effects of extracellular matrix components due to impaired PHEX function.^(6)^

Adult patients may present with osteomalacia, fractures, pseudo-fractures, enthesopathies, osteoarthritis, bone and joint pain, and hearing loss. They are also prone to secondary and tertiary hyperparathyroidism, as well as cardiovascular and metabolic disorders.^(5,7)^

Diagnosis of XLH is multifactorial and based on clinical, laboratory, and radiological findings, as well as family history. Biochemical tests show low serum phosphate levels; elevated levels of FGF23 and alkaline phosphatase (ALP), normal serum calcium and parathyroid hormone (PTH) levels, and low or normal calcitriol levels. The TmP/GFR ratio, which corresponds to the maximum tubular phosphate reabsorption rate in relation to the glomerular filtration rate, is also low in XLH.^(2)^ Bone radiographs show bone deformities, radiopaque growth lines, and widening of metaphyseal zones. Molecular sequencing of the *PHEX* gene is another tool that, when available, is useful for confirming the diagnosis, determining themutation status of family members, and providing genetic counseling. This study describes the clinical, laboratory, radiological, and molecular findings of three pediatric patients with XLH treated with the monoclonal antibody burosumab and followed up at the Pediatric Specialty Clinic of *Hospital Israelita Albert Einstein* in São Paulo, Brazil.

## CASE REPORT

The first two patients are siblings: Patient 1 is an 18-year-old Caucasian male, born at term by cesarean section after a complicated pregnancy. His birth weight was 2.9kg, 47cm, and the Apgar scores were normal. At 6 years of age, he was referred to the outpatient clinic by his pediatrician due to progressively worsening bowing of the legs, which had been observed since 18 months of age. The family history was negative for parental consanguinity. His mother presented with significant short stature (138cm), osteomalacia, and bowed legs (Figure 1). On physical examination, the patient's height was 102.7cm (-3 SD), and weight was 16kg (-2 SD) (Figure 2), with noticeable bowing of the legs. His dentition appeared normal.

**Figure 1:**
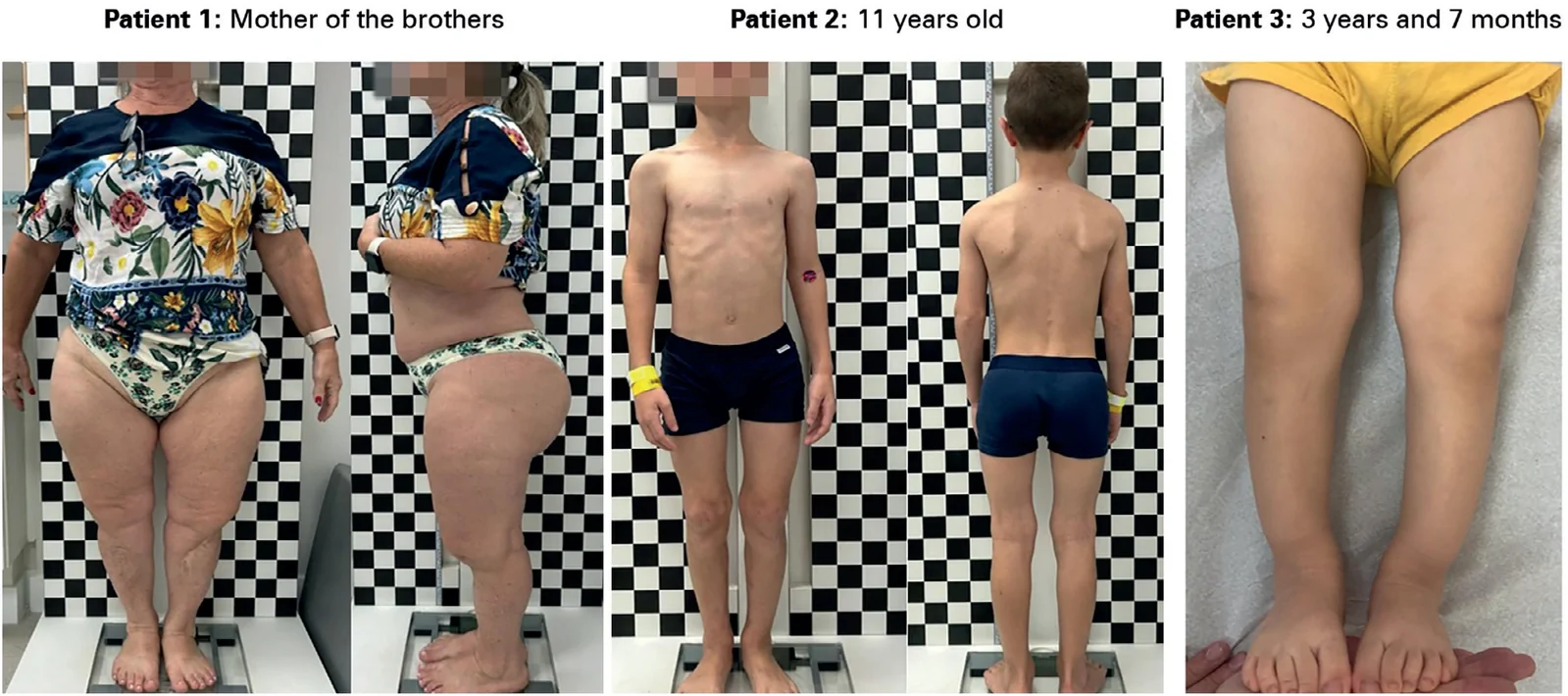
Current photos of the brothers’ mother, patient 2 and patient 3

**Figure 2:**
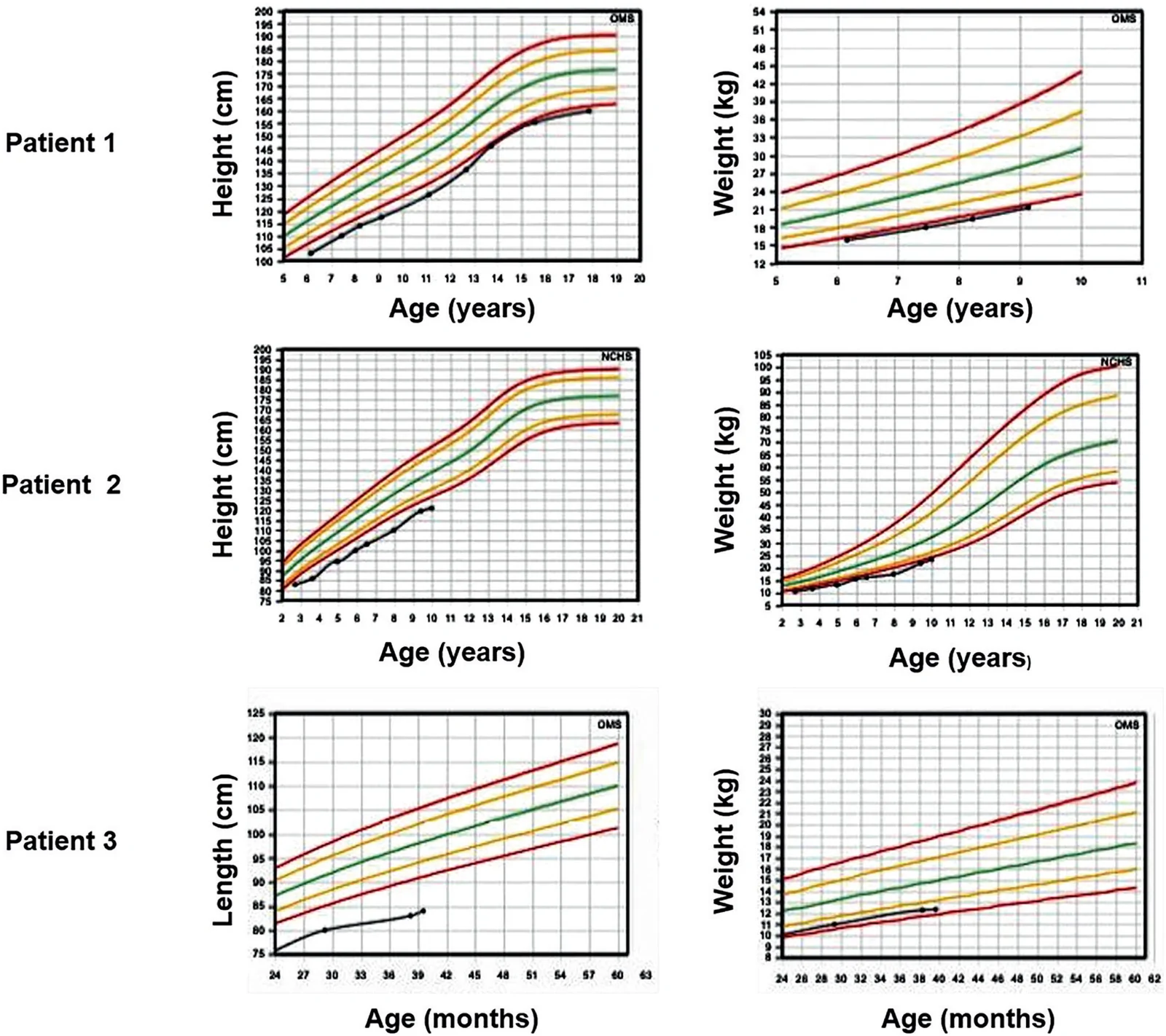
Growth chart

Initial laboratory evaluation revealed hypophosphatemia and increased ALP levels. Serum calcium, vitamin D levels, PTH, blood gas analysis, and serum electrolytes were all normal, as was the initial renal ultrasound. The 24-hour urine test showed hyperphosphaturia (Table 1) and normal calciuria, with a urinary calcium/creatinine ratio of 0.02 (reference: <0.2 for children older than 5 years). Serum FGF23 levels could not be assessed because this test was not available at that time.

**Table 1:** Clinical, biochemical, and radiological findings at diagnosis and after one year of treatment with burosumab in three patients with X-linked hypophosphatemia

Variable	Patient 1		Patient 2		Patient 3	
	Diagnosis 6 years	After 1 year of treatment with Burosumab 30mg (Age: 19 years)	Diagnosis 17 months	After 1 year of treatment with Burosumab 20mg (Age: 11 years)	Diagnosis 7 months	After 1 year of treatment with Burosumab 10mg (Age: 3 years 7 months)
Serum Ca (mg/dL)	9.8 (8.6-10)	na	10.5 (8.6-10)	na	10.4 (8.6-10)	na
Serum P (mg/dL)	2.8 (4.0-7.0)	3.1 (2.5-5.6)	2.6 (3.0-6.9)	3.7 (4.0-5.6)	2.7 (4.0-6.5)	3.4 (4.4-6.2)
ALP (U/L)	419 (150-380)	127 (55-149)	383 (145-320)	320 (129-417)	699 (<600)	343 (104-345)
PTH (pg/mL)	35 (15-65)	na	50.8 (15-65)	na	25.6 (18-88)	na
25(OH) Vitamin D (ng/mL)	45 (20-60)	na	23.4 (20-60)	na	23.7 (20-60)	na
Calcitriol (pg/mL)	40 (31-87)	na	40.5 (31-87)	na	na	na
Venous blood pH	7.38 (7.32-7.43)	na	7.39 (7.32-7.43)	na	7.4 (7.32-7.43)	na
HCO^3^- (mEq/L)	26.2 (22-29)	na	24.5 (22-29)	na	24.8 (22-29)	na
Serum creatinine (mg/dL)	0.3 (0.7-1.2)	0.6 (0.7-1.2)	0.33 (0.7-1.2)	0.36 (0.32-1.0)	0.18	0.31
Urinary P - 24h (mg/24h)	1.55 (0.4-1.2)	na	na	na	na	na
Urinary creatinine - 24h (mg/24h)	0.5 (0.17-1.41)	na	na	na	na	na
Urinary P - isolated (mg/dL)	na	100 (40-278)	1773	25.6 (40-278)	4.0	12.9
Urinary creatinine - isolated (mg/dL)	na	51	40.8	31.8	11.26	25
TmP/GFR (mg/dL)	na	3.09 (2.6-3.8)	2.47 (3.25-5.51)	3.7 (3.0-5.08)	2.7 (3.5-5.12)	3.4 (3.25-5.51)
**Renal ultrasound**	**Normal**	**Nephrocalcinosis: stable**	**Normal**	**Nephrocalcinosis: stable**	**Normal**	**Normal**
	**Patient 1**		**Patient 2**		**Patient 3**	
Age duration treatment with oral phosphate	6 years - 18 years		17 months - 10 years		8 months - 16 months	
Need for surgery	Yes		Yes		No	
PHEX mutation type	na		Nonsense mutation, c.1778_1779del (p.Tyr593*)		Hemizygous intragenic deletion of exon 6 (ChrX:22,090,352-22,090,611)	

* stop codon.

na: not available; in red: values outside the reference; Ca: calcium; P: phosphorus; PTH: parathyroid hormone; ALP: alkaline phosphatase; TmP/GFR: ratio of tubular maximum reabsorption of phosphate to glomerular filtration rate; U: international units.

Radiographs of the femur and tibia showed bilateral medial deviation of the femoral and tibial metaphyses, with increased valgus of knees and ankles, widening of all metaphyses with epiphyseal irregularity, suggesting metabolic bone disease (Figure 3).

**Figure 3:**
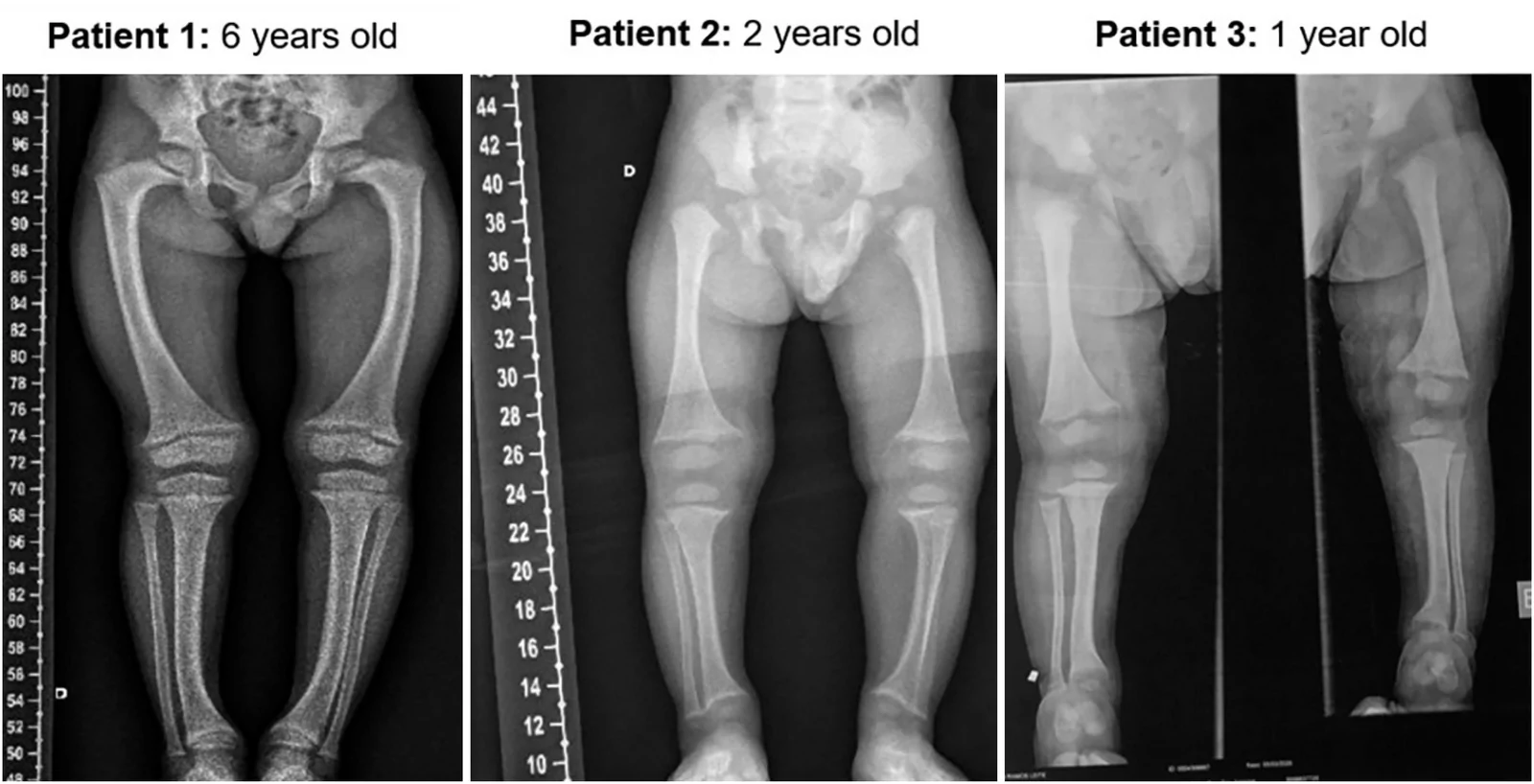
Radiographs of the three patients during treatment with phosphate salts

Initially, XLH was suspected, and the patient was started on oral phosphate salts at a dose of 30-60mg/kg/day of elemental phosphate and 25*µ*g/day of calcitriol. The oral phosphate dose was adjusted according to serum phosphate levels, but maintaining serum phosphate, alkaline phosphatase, and PTH within the normal range during conventional treatment was very difficult. Over subsequent years, the patient was followed by a multidisciplinary team that included his pediatrician, endocrinologist, orthopedic surgeon, and nephrologist. Serial renal ultrasounds revealed a slight nephrocalcinosis, but renal function remained normal. Orthopedic treatment consisted of several femoral osteotomies and guided growth surgeries aimed at realigning the bone deformity. Realignment of the proximal femur was achieved with a valgus osteotomy, while the distal femur and both the proximal and distal tibia were treated with "eight plates" fixed on the convexity of the bone at the growth plate level until bone alignment was achieved. The metal plate was then removed, and the bone was allowed to grow freely.

After two years of treatment, renal ultrasound revealed a slight nephrocalcinosis, but renal function remained normal. The patient received oral phosphate salts until eighteen years of age, when treatment was changed to burosumab, following the molecular confirmation of XLH in his younger brother. Burosumab is a monoclonal antibody against FGF23 that is expected to restore normal phosphate reabsorption by the kidney and increase calcitriol production. After two months of treatment, serum phosphaturia normalized, which was demonstrated by normal renal tubular phosphate reabsorption (TmP/GFR) and normal serum phosphate and ALP (Table 1). He is currently 19 years old, his legs are almost straight, and he has reached a height of 160cm (Table 2). Patient 2 is eight years younger than his brother. He was born by cesarean section, without complications, with a birth weight of 2.6kg and length of 48cm. Hypophosphatemia was detected by the pediatrician before 12 months of age, but he was started on oral phosphate salts (30–60mg/kg/day of elemental phosphate) and calcitriol (25*µ*g/day) at 17 months of age. Table 1 shows the laboratory and ultrasound results at the time of diagnosis: hypophosphatemia, hyperphosphaturia, low TmP/GFR, increased ALP, and normal renal ultrasound findings. Maintaining normal serum phosphate levels with this medication was very difficult, and his legs became bowed when he started walking (Figure 3). He underwent the same orthopedic treatment as his brother, with satisfactory results. Nephrocalcinosis was also detected after a few years of active disease, but it remained stable, and renal function was preserved. He is currently a healthy boy, with short stature and bowed legs (Figures 1 and 2).

A next-generation sequencing (NGS) panel of *13* genes for hypophosphatemic rickets was performed at 7 years of age, and the results revealed the inactivating c1778_1779del mutation in the *PHEX* gene, generating a stop codon and a truncated PHEX protein (p.Tyr593*). After molecular confirmation of dominant XLH, he began treatment with burosumab at a starting dose of 0.8mg/kg of body weight, which had to be gradually adjusted until normalization of TmP/GFR and ALP levels. Serum phosphate remained slightly low (Table 1).

Patient 3 is a 4-year-old Caucasian boy, born at term by cesarean section, with a birth weight of 2.930kg and length of 47.5cm, after an uncomplicated delivery. There was no history of consanguinity in this family, and his mother had short stature (144cm) and a clinical and radiological diagnosis of XLH since childhood. The first biochemical study, performed at 4 months of age, revealed low TmP/GFR and elevated ALP levels (Table 1). His diet was then changed to a phosphorus-rich formula, which he consumed for several months. Follow-up laboratory tests revealed persistent hypophosphatemia and hyperphosphaturia, and his growth rate decreased. At 8 months of age, the patient began taking oral phosphate salts at a dose of 30 to 60mg/kg/day of elemental phosphate and 25*µ*g/day of calcitriol, but hypophosphatemia persisted, and rachitic changes in the long bones appeared, such as lateral widening of metaphyseal regions and bone demineralization (Figure 3). When he began to walk, his legs began to bow, and he frequently complained of pain in the lower limbs. This patient also developed abnormal dentin and was prone to endodontic infections (Figure 4).

**Figure 4:**
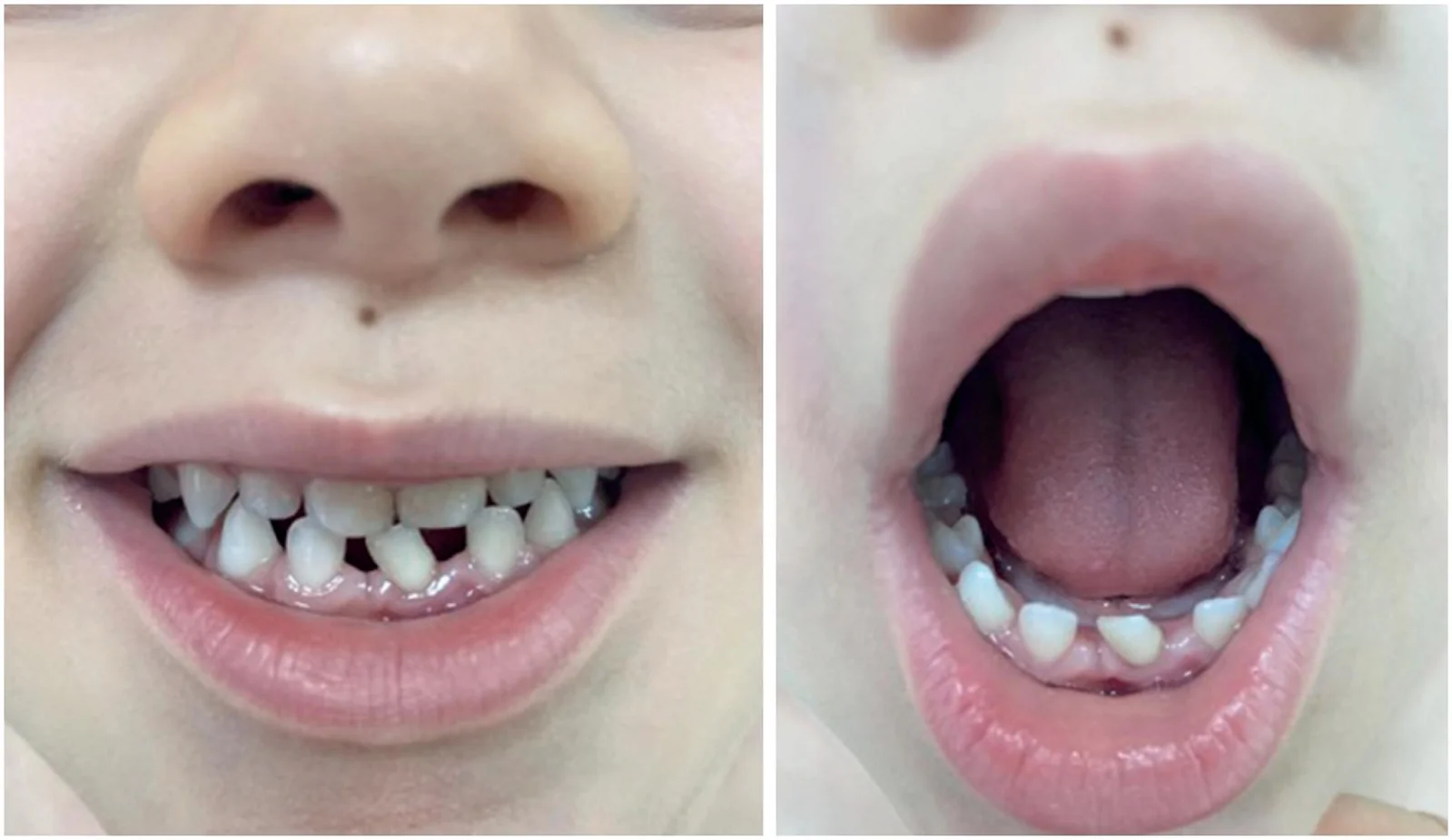
Patient 3 and his dental alteration and gingivitis

A NGS panel for hypophosphatemic rickets revealed a hemizygous deletion in exon 6, encompassing the region ChrX: 22.090.352_22.090.611. His mother underwent *PHEX* gene analysis using the MLPA technique (*multiplex ligation-dependent probe amplification*), and the same deletion was found, confirming the diagnosis of XLH.

Treatment with burosumab was initiated at 16 months of age, and since then, he has stopped complaining of leg pain. Dental abnormalities persist, and he requires frequent follow-up with a dentist to prevent or treat dental abscesses. Alkaline phosphatase and renal phosphate reabsorption have normalized, but serum phosphate remains low, although close to the lower limit of normal (Table 1). Currently, at 3 years and 7 months of age, his growth rate has accelerated to 8cm/year; therefore, he may be beginning to experience growth recovery, which will need to be closely monitored. His orthopedic follow-up has been frequent, and there has been no indication for orthopedic surgery so far.

## ETHICAL ASPECTS

Written informed consent was obtained from the patients and their legal guardians for the retrospective use and publication of anonymized clinical, laboratory, and imaging data. Ethics approval for this case report was granted by the Institutional Ethics Committee of *Hospital Israelita Albert Einstein* (CAAE: 81440124.7.0000.0071; # 6.987.493, SGPP: 6987493).

## DISCUSSION

Clinical, radiological, laboratory, and molecular findings, together with treatment approaches, are analyzed in three patients with XLH, all of whom harbored inactivating mutations in the *PHEX* gene.

The main clinical symptoms of XLH result from hypophosphatemia secondary to hyperphosphaturia, detected by reduced phosphate reabsorption (TmP/GFR). Young children may exhibit craniosynostosis and increased intracranial pressure due to abnormal ossification of the cranial vault and may require neurological and neurosurgical care. Additional findings include frontal bossing and rachitic rosary. At the end of the first year, with the onset of walking, a varus deformity of the lower limbs appears. Unfortunately, this finding is commonly misinterpreted as physiological bowing of the legs, which can delay diagnosis. Skeletal hypomineralization and enlarged rachitic growth plates lead to progressive varus and valgus deformities over the years. Torsional deformities can result in impaired gait patterns, such as intoeing. Muscle weakness and musculoskeletal pain further affect motor function. As the child grows, height is affected by lower limb deformities, resulting in short stature, because the spine grows relatively normally, disproportionate body shape is a common feature.^(1,2,8,9)^ Dentin hypomineralization predisposes patients to endodontic infections, which may occur despite proper dental care.^(6)^

Despite his severe disproportionate short stature, bowed legs, and a mother with the same phenotype, Patient 1 received a late diagnosis of X-linked hypophosphatemia; consequently, treatment with oral phosphate and calcitriol was started late, at 6 years of age. In contrast, when his brother was born, the pediatrician, aware of the older brother's disease, initiated treatment with phosphate salts and calcitriol as soon as hypophosphatemia and elevated ALP levels were detected.

Although treatment was initiated at 8 months of age, early intervention did not prevent disease progression, but may have attenuated its severity, as previous studies have shown that earlier treatment initiation is associated with slower progression of rickets.^(9,10)^ A similar pattern was observed in Patient 3, who developed progressive skeletal hypomineralization and bone deformities despite early treatment with phosphate.

In addition to clinical, biochemical, and radiological examinations, molecular studies represent an additional tool that, when available, is useful for confirming the diagnosis of XLH, determining the mutation status of family members, and providing genetic counseling. More than 800 variants of the *PHEX* gene have been identified in the Human Genome Mutation Database. These variants may include nonsense, missense, splicing, or frameshift, with penetrance that appears to be close to 100% within the first year of life.^(11,12)^ Disease severity varies significantly among family members, and there is controversy regarding whether XLH manifests more severely in males. If so, a possible explanation could be the occurrence of the mutation

on the inactivated X chromosome, as females exhibit partial inactivation of one of their X chromosomes.^(13,14)^

Two of the families described here underwent molecular sequencing of the *PHEX* gene. Among siblings, the younger brother underwent a multigene NGS panel that included the *PHEX* gene. Final analysis revealed a hemizygous nonsense variant in *PHEX*, c.1778_1779del (p.Tyr593*), creating a premature stop codon and resulting in a loss-of-function protein. Loss-of-function variants in *PHEX* are known to be pathogenic. This variant is absent from population databases and has been previously reported in affected individuals.^(4,14-16)^ The Affected mother and her older son did not undergo molecular confirmation; however, their clinical and laboratory findings are fully consistent with XLH. The mother is severely affected, with bowed legs and a height of 138cm. Ongoing discussion in the literature addresses whether XLH manifests more severely in males. One possible explanation for this variability may involve random X-chromosome inactivation in females, resulting in mosaic expression of wild-type and mutant *PHEX* alleles in approximately equal proportions. The relative expression of the mutant allele in relevant tissues may influence clinical severity.

Patient 3 has a hemizygous intragenic deletion in the *PHEX* gene, comprising exon 6 and spanning approximately ChrX:22.090.352– 22.090.611, inherited from his mother, who is heterozygous for the same variant. This variant was not found in any databases and may result in a substantial alteration in protein structure, potentially affecting its function.

Conventional treatment of XLH with active vitamin D and oral phosphate has long been challenging because of poor intestinal absorption of phosphate salts, intestinal mucosal irritation, the short half-life of phosphate salts (requiring administration in divided doses four to six times daily), and their unpleasant taste. These limitations are clinically relevant, as they directly compromise treatment adherence. When serum phosphate levels are normalized through high-dose therapy, increased phosphate availability may result in adverse effects such as hyperparathyroidism and nephrocalcinosis.^(2,17)^ For these reasons, achieving an adequate balance between optimization of serum phosphate and ALP levels and minimization of adverse effects remains difficult and is rarely fully accomplished, allowing rickets to progress as children grow. All three patients showed unsatisfactory responses to oral phosphate therapy, likely related to the limitations described above. The balance between improvement of skeletal manifestations and prevention of adverse effects was never achieved. The siblings developed nephrocalcinosis after two years of oral phosphate treatment, and skeletal abnormalities persisted. Orthopedic surgeries partially corrected bone deformities. This clinical scenario led to the initiation of burosumab therapy, a monoclonal antibody targeting FGF23.

Because conventional treatment for XLH with oral phosphate salts and calcitriol is often unsuccessful, efforts have focused on therapies that directly target the underlying pathophysiology of the disease, namely excess FGF23 activity. Burosumab, a monoclonal antibody that neutralizes circulating FGF23, improves renal phosphate reabsorption and increases calcitriol synthesis, thereby restoring phosphate homeostasis. Clinically, this results in improvements in rickets, skeletal manifestations, and growth. However, dental abnormalities often persist because they appear to result from intrinsic defects in the dentin matrix related to impaired PHEX function rather than systemic phosphate dysregulation.^(6,10,18)^

Burosumab was approved by the FDA in 2018 for the treatment of XLH following a phase 3, randomized, active-controlled, open-label study that included 61 moderately affected children aged 1 to 12 years. Eligible patients were randomly assigned to receive subcutaneous burosumab or conventional therapy, and the interventions lasted 64 weeks. Ultimately, the study demonstrated the superiority of burosumab over conventional therapy in terms of growth and radiographic improvement, wich was the primary endpoint of the study.^(10)^ Subsequently, the same group reported 160 weeks of follow-up data for these children and concluded that burosumab had sustained efficacy and was well-tolerated.^(18)^ Another publication from this group presented a post hoc analysis of the efficacy and safety of burosumab in children younger than 5 years who participated in the same 64-week open-label phase 3 study, The authors concluded that, in this population of young children, burosumab was associated with better outcomes than conventional treatment, including improvements in rickets, lower limb deformities, growth, and, ALP levels.^(19)^

Based on these data, treatment was switched to burosumab in all patients. Phosphate salts and calcitriol were discontinued, and burosumab was initiated according to the recommended dosing regimen.^(20)^ After approximately two months, ALP levels and phosphate excretion normalized, although serum phosphate remained slightly below the lower limit of normal in Patients 2 and 3. Patient 1, who initiated treatment at 16 years of age, showed rapid normalization of serum phosphate. Radiological improvement of rickets was observed in the siblings despite borderline phosphate levels, and treatment adherence improved because of the greater convenience of the regimen.

These findings are consistent with those of a 2023 British study in which the authors conducted a retrospective review of burosumab treatment outcomes in a cohort of 54 children with XLH attending the same endocrinology service. They assessed growth and biochemical markers of bone health in these children and observed that most of them (41 children) normalized their ALP levels, whereas only half (27 children) achieved normal plasma phosphate concentrations. Despite this disparity, no differences in growth were found between the groups with normal and abnormal phosphate levels. These results led the authors to question current recommendations for adjusting burosumab doses to normalize phosphate levels. Instead, they hypothesize that ALP levels may be more relevant than phosphate levels for dosing this medication. However, a prospective study with a larger number of patients would be needed to confirm their hypothesis.^(21)^

## CONCLUSION

Genetic testing, guided by clinical and radiological findings, was crucial for establishing a definitive diagnosis of X-linked hypophosphatemic. Diagnostic confirmation supported the initiation and long-term use of burosumab therapy. All patients showed a favorable clinical and biochemical response despite persistently borderline serum phosphate levels. Improved treatment adherence, largely attributable to ease of administration, likely contributed to therapeutic success.

## Data Availability

The underlying content is contained within the manuscript.
